# Stroke survivors' perspectives on decision-making about rehabilitation and the prospect of taking recovery-promoting drugs: A qualitative study

**DOI:** 10.1016/j.rcsop.2023.100297

**Published:** 2023-06-28

**Authors:** Nerida Firth, Kathryn S. Hayward, Julie Bernhardt, Robin Ray, Ruth N. Barker

**Affiliations:** a College of Medicine and Dentistry, James Cook University, Townsville, Australia; b Departments of Physiotherapy and Medicine, University of Melbourne, Melbourne, Australia; c Florey Institute of Neuroscience and Mental Health, University of Melbourne, Melbourne, Australia; d NHMRC Centre for Research Excellence in Stroke Rehabilitation and Brain Recovery, Melbourne, Australia; e College of Healthcare Sciences, James Cook University, Cairns, Australia

**Keywords:** Stroke, Rehabilitation, Recovery, Pharmacotherapy, Medication, Drug

## Abstract

**Objectives:**

To investigate factors which influence stroke survivors' decision-making about their rehabilitation and the prospect of taking recovery-promoting drugs, to enhance their recovery.

**Methods:**

Seventeen stroke survivors who had undertaken stroke rehabilitation, and three spouses, participated in focus groups and individual interviews in northern Queensland, Australia. Inductive thematic analysis of the interview data was conducted in accordance with Braun and Clarke's six-phase process.

**Results:**

Two specific, pivotal decision points during participants' stroke recovery process were identified: 1) overall, when deciding what rehabilitation they would undertake and hypothetically what recovery-promoting drugs they would take, and 2) on a daily basis, when deciding whether to participate in rehabilitation and take recovery-promoting drugs on any given day. Six themes which described factors influencing their decision-making were: ‘My options for rehabilitation and recovery-promoting drugs’; ‘The costs of rehabilitation and recovery-promoting drugs’; ‘My recovery goals’; ‘What I can deal with today’; ‘The people my rehabilitation and recovery-promoting drugs affect’; and ‘Fitting rehabilitation and recovery-promoting drugs into my life.’ These themes were applicable at either one or both of the identified decision points.

**Conclusion:**

Factors that influence stroke survivors' decision-making, overall and on a day-to-day basis, need to be considered to ensure they can make the best decisions for themselves to achieve their full recovery potential. Understanding the conditions under which a stroke survivor would take a recovery-promoting drug will contribute to the development of dosing protocols to which stroke survivors could adhere.

## 1 Introduction

Stroke survivor preferences with regard to the costs, risks and inconveniences associated with rehabilitation interventions to improve their recovery play an important role in whether they choose to adhere to the intervention.^1^, ^2^, ^3^ For example, the cost of transport to the rehabilitation clinic, the risk of falling while transiting and the inconvenience of waiting to be collected after an appointment are all factors which may affect adherence. Such factors must be considered with the advent of recovery-promoting drugs (RPDs), defined as medications that promote motor recovery post-stroke.^4^^,^^5^ To date, drugs investigated for recovery-promoting potential have included amphetamine, cerebrolysin, citalopram, fluoxetine, lithium and selegilene.^6^ The conditions under which stroke survivors would commit to taking RPDs as prescribed are largely unknown. Understanding their preferences, and the factors that underpin them, is necessary if clinicians are to support stroke survivors to make informed decisions about RPDs that they would take precisely as prescribed to maximise their recovery.^3^ Furthermore, accommodating stroke survivors' preferences for physical and behavioural rehabilitation interventions (referred to as rehabilitation hereafter) and RPDs, alone or in combination, has potential to improve trial fidelity and translation into real-world practice.^7^ This knowledge could contribute to the development of robust clinical guidelines for RPD use to promote stroke recovery.

Stroke survivor preferences are influenced by many and varied factors, each of which hold varying importance to each individual.^1^^,^^3^ Discrete choice experiments allow investigation into the interplay of such factors that may influence a person's preference by examining the trade-offs respondents are willing to make between different key attributes in decision-making.^8^ While several DCEs have been undertaken to investigate stroke survivors' preferences for rehabilitation and for medications to reduce stroke risk,^3^^,^^9^, ^10^, ^11^, ^12^ none have been conducted to explore stroke survivors' decision-making about their rehabilitation and prospect of taking recovery-promoting drugs. When developing a robust discrete choice experiment, the key factors influencing decision-making must be known.^13^ Because RPDs have only been used in clinical trials, finding stroke survivors with experience taking them is near impossible. Until such time as RPDs are routinely available or prescribed, exploring how stroke survivors make decisions about their participation in rehabilitation will provide important insight into these factors. By posing the hypothetical question of whether they would take RPDs, and under what circumstances, stroke survivors can draw on their lived experience of making decisions to promote their recovery and provide their best estimation of which factors would influence their decision-making. Therefore, the aim of this study was to investigate factors which would influence stroke survivors' decision-making about their rehabilitation, and the prospect of taking RPDs, to enhance their recovery.

## 2 Methods

### 2.1 Design

A qualitative descriptive methodology was chosen to enable rich, detailed descriptions of participants' decision-making processes regarding rehabilitation and RPDs to promote their recovery, to be obtained in their own voice.^14^^,^^15^ Six focus groups and five individual interviews were conducted over five months (May to September 2019). Focus groups enabled participant interaction, facilitating connections based on mutual experiences.^16^^,^^17^ Individual interviews provided a private, safe opportunity for personal, detailed accounts of their experience.^15^ Vast geographical distance between participants' place of residence meant that their location and timing of recruitment determined when FGs or interviews were held. Participants were allocated to focus groups or individual interviews according to participant preference and convenience. The number of focus groups and individual interviews conducted was determined at the point of data sufficiency, when no new codes or themes were identified during data analysis.^18^ This study was approved by James Cook University Human Research Ethics Committee (Approval number H7620).

### 2.2 Participants

Participants were purposively selected to ensure maximum variation in terms of demographics, socioeconomics, and stroke experience.^19^ Recruitment across northern Queensland (Australia) occurred via social media (Facebook and Twitter); media releases, radio news bulletins and radio interviews; and information flyers displayed on community noticeboards and at medical and allied health clinics, consultant neurologist clinics and community rehabilitation centres. Participants were required to be at least 18 years old; previously diagnosed with stroke and had undertaken stroke rehabilitation (inpatient, outpatient, community or home rehabilitation) at any point post-stroke; and could provide consent. Potential participants were excluded if they had history of other neurological conditions or injury (such as Parkinson's Disease). Informed written consent was received from all participants.

### 2.3 Data collection

A semi-structured question guide was developed for use in focus group and individual interviews based on the scientific literature and discussions with experts in the stroke rehabilitation field.^16^^,^^20^ Questions were open-ended to gain subjective meaning and achieve the aims of the study.^16^ The resulting topic guide was piloted with stroke research peers, who provided feedback on the language used. The topic guide contained six domains including four separate topics regarding inpatient and outpatient stroke rehabilitation experience and decision-making about taking RPDs (supplied as Supplementary Material). Each domain contained at least one question, with follow-up questions and probes to facilitate robust conversation. Interviews were conducted by the primary researcher (NF). There was no strict adherence to the style and type of questioning beyond the key questions.^16^^,^^20^ As the primary researcher was a novice researcher, the first two focus groups were co-facilitated by a second researcher (CS). All focus group and individual interviews were audio-recorded (with consent) and transcribed by a professional transcription service; transcripts were checked against the audio recordings for accuracy.^17^^,^^20^

### 2.4 Data analysis and reporting

Transcripts, interview summaries and team debriefing notes were all included as data and uploaded into NVivo qualitative analysis software, QSR International Pty Ltd. Version 12.^17^ An iterative approach to continued data collection and analysis was undertaken, occurring concurrently to obtain thick, rich data.^20^^,^^21^ Inductive thematic analysis was undertaken in accordance with Braun and Clarke's six-phase process.^22^ First, transcripts were read several times alongside the audio recordings to enable NF to become familiar with the data and obtain a sense of the whole.^20^^,^^22^ An initial list of codes was compiled at a mostly latent level, although some semantic analysis of content occurred where deemed appropriate and interesting.^22^ Codes were then sorted into themes with several thematic maps developed, and the relationships between the codes and potential themes discussed within the research team. A second researcher (RR) undertook coding of one interview data set, which was checked against the coding of NF, for confirmability and credibility of the data analysis.^14^^,^^16^ The remainder of the data set was coded. The research team met throughout the process to discuss interpretations, with recoding occurring as appropriate upon reflection and a revisiting of the data.^14^^,^^21^^,^^23^ A thematic map was developed which was then considered in the context of the entire data set and whether it accurately represented the meanings evident in the data set as a whole. To optimise transferability, rich and detailed descriptions of the study design, methods and results are reported in this manuscript. In doing so, readers are provided with an opportunity to compare and contrast their own contexts with the context of this research, allowing them to determine whether these research findings are transferable to their own situation.^17^

### 2.5 Research team and reflexivity

The primary researcher and interviewer/focus group facilitator (NF) is a pharmacist with experience working in a community-based neurological rehabilitation centre in regional Australia and has training in qualitative research methods. NF worked with an experienced team of three stroke rehabilitation physiotherapists/researchers (RB, KH, JB) and a health professional academic specialising in qualitative research methods (RR). The primary researcher held no prior relationship with participants. It was made clear that NF was a pharmacist seeking to learn more about factors influencing stroke survivors' decision-making about stroke rehabilitation options, with a particular interest in recovery-promoting drugs.

## 3 Results

### 3.1 Participants

The final 20 participants were drawn from 25 potentially eligible participants, one of whom withdrew before interview due to poor health and four who did not respond after the screening process. Of the 20 participants, 17 were stroke survivors and three were spouses of stroke survivors who attended to support and assist with communication. Participant demographics are summarised in Table 1. Duration of focus group and individual interviews ranged between 61 and 94 min. It is also worth acknowledging that the data were captured pre-pandemic and that with the increased use of telerehabilitation some of these views may have changed.

**Table 1:** Participant characteristics.

Stroke survivors = 17					
Gender	Age^+^	Time since stroke^+^	Affected side (body)	Rehabilitation service attended	Therapy undertaken
Male = 9	Under 40 yrs. = 1	Mean = 8 yrs. 7mo	Left = 11	Hospital rehabilitation = 10	Physiotherapy = 3
Female = 8	40-59 yrs. = 5	Range: 1 yr – 42 yrs	Right = 5	Subacute rehabilitation = 3	Occupational therapy = 5
Other = 0	60 yrs. and over = 11		Both = 1	Community rehabilitation = 9	Speech pathology = 5
				Rehabilitation at home = 1	Exercise physiology = 1
				Private allied health service = 4	Osteopathy = 1
					Neuropsychology = 1
					Psychology = 1


Spouses/support person = 3					
Gender	Age^+^				
Male = 2	Under 40 yrs. = 0				
Female = 1	40-59 yrs. = 0				
Other = 0	60 yrs. and over = 3				

^+^At time of screening.

Abbreviations: yr = year/s; mo = month/s.

### 3.2 Themes

**Fig. 1:**
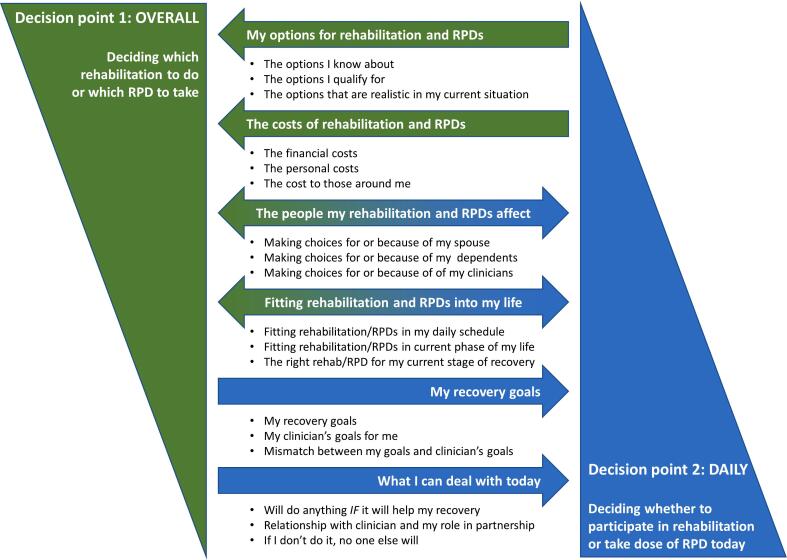
Factors which influence stroke survivors' decision-making about their rehabilitation, and the prospect of taking RPDs: six main themes.

Participants discussed a wide range of reasons why and how they made decisions to participate in rehabilitation and hypothetically, why they would take RPDs. Whilst the decisions each participant made were personal and unique, many similar factors influenced their decision making. Six strong themes were identified from the data. It became apparent that these themes were relevant at two specific decision points in participants' recovery process (Fig. 1).

The first decision point occurred when participants chose the type of rehabilitation they would undertake, or hypothetically, when they would agree to start taking RPDs. At this point their decision appeared to be influenced by: ‘My options for rehabilitation and RPDs’ and ‘The costs of rehabilitation and RPDs.’ The second decision point occurred on a daily basis, when participants decided whether to actively engage in rehabilitation that day and to what extent, or whether to take a prescribed dose of RPD. This decision point appeared to be influenced by ‘My recovery goals’ and ‘What I can deal with today.’ A further two themes were applicable at both decision points: ‘The people my rehabilitation and RPDs affect’ and ‘Fitting rehabilitation and RPDs into my life.’

*Decision point 1: Deciding which rehabilitation to do or which RPD to take.*

*‘My options for rehabilitation and RPDs.’*

Knowing what options were available clearly influenced decision-making, however this varied widely between participants. Some were aware of the options available to them, while others learned of new, different options as time passed.

“But I think some of the things I've done, have really, really helped me. And there are things that others have really helped them that I don't know about.” [Female, 40-59 yr, 7 yrs. post stroke].

Some participants knew of options but believed they were ineligible.

“I have had no rehab at all from anybody or any organisation [since returning from hospital] because I live in [small rural town].” [Male, 60 yr and over, 5 yrs. post stroke].

Whether excluded due to real or perceived barriers, participants believed that some rehabilitation options were not feasible for them. Some reported they could not organise consistent transport, or waiting lists were too long. Others believed it was too late and they were ineligible when they eventually learned of rehabilitation services.

When discussing RPDs, participants said they would be willing to give anything a go, suggesting they would ‘probably have no hesitation’ to take an experimental or ‘miracle’ drug. Some participants had explored options for stem cell treatments and natural therapies, although did not follow through to join trials.

*‘The costs of rehabilitation and RPDs.’*

Participants discussed the effort, losses and sacrifices associated with rehabilitation and RPDs after stroke. These costs were both financial and non-financial.

“My gym program was set up by a physio from [community-based rehabilitation service]. He came with me the first time, he set up a program, but if you're on $AU250 a week, you can't afford $AU14 a week to go to a gym, and $AU4 a day to go to a pool.” [Male, 40-59 yr, 11 yrs. post stroke].

Personal costs discussed by participants included discomfort experienced; inconvenience and burden of rehabilitation and RPDs for both them and their families; and time that attending rehabilitation would take out of their lives.

“At that time [participant's parents] were self-employed, they were fortunate enough to be a little bit more flexible… in the long term, it impacted on their financial income as well. … there was a lot of waiting round for them.” [Male, under 40 yr, 14 yrs. post stroke].

When discussing RPDs, participants spoke of justifying the expense of a medication with the value gained from taking it.

“I wouldn't have any hesitation if back then the epilepsy drug cost $60 a month. If it controlled my seizures [after the stroke] that would … be sufficient, because I'm getting a result that I want.” [Male, under 40 yr, 14 yrs. post stroke].

*Decision point 2: Deciding whether to participate in rehabilitation or take dose of RPD today.*

*‘My recovery goals.’*

Their personal recovery goals influenced whether a participant would actively engage in their rehabilitation that day. The rehabilitation plan was shaped by their goals, and these goals motivated the participants to persevere.

“What drives me is I still need a goal.” [Female, 40-59 yr, 7 yrs. post stroke].

The rehabilitation clinician's goals for the session also influenced participants' decisions to actively engage. When participants understood the clinician's goals, they were more motivated.

“They actually came out to my shed… looked at everything I had… and what I was trying to do… the next time I showed up for an OT session she pulled out a tin full of nuts and bolts… I had to put the nuts and bolts and washers and all that sort of stuff together. And you know, that all made sense.” [Male, 60 yr and over, 1 yr post stroke].

When participants could not align the clinician's goals for the rehabilitation session with their personal recovery goals, they expressed dissatisfaction with the service, disillusionment with the rehabilitation process and distrust of their clinician's capacity.

“One of [my goals] was to get up off the floor… I'd work with the same person for a couple of weeks… but then you'd go in next time, someone different, and they go, ‘we'll do this and we'll do that,’ and then there go[es] all the work you've just built up.” [Female, 40-59 yr, 1 yr post stroke].

The decision to take RPDs was often influenced by the impact on participants achieving their goals.

“I hadn't noticed being drowsy [while on a prescribed medication] but I was just worried about getting drowsy you know, because I wanted my license back and driving again…” [Male, 60 yr and over, 1 yr post stroke].

Achieving rehabilitation goals despite experiencing side-effects due to RPDs was considered worthwhile in some instances. A participant used a hypothetical example of a RPD that could cause hair loss but enhanced recovery of hand function during rehabilitation.

“Well, I can't put on headphones. Because they require two hands. …. If I had my hand back, I would be able to get on a wig.” [Female, 60 yr and over, 22 yrs. post stroke].

*‘What I can deal with today.’*

How they felt and what they could deal with that day influenced participants' decisions to engage in rehabilitation or take RPDs. Some participants reported that they would ‘do anything’ or ‘have a crack,’ particularly ‘if it's going to help me.’

Positive relationships with clinicians, and believing the clinicians were invested in their recovery motivated participants to actively engage in their rehabilitation sessions.

“I was very committed to my physios at the hospital, and I think some of those more personal relationships, more than anything, kept me there…” [Female, 40-59 yr, 1 yr post stroke].

Conversely, some participants who worked with different clinicians at each rehabilitation session expressed their dissatisfaction. They did not want to ‘explain [their story] all again’ to a new clinician. If the new clinician suggested something different or deviated from the set rehabilitation plan some participants felt the clinician was experimenting.

“You've got a program… you're working towards something… you feel like you're going in the one direction. Every day you're doing more or better at it, then you get a different person that works with you, and they go, I just want to try this with you…. I'm like, ‘I'm not just here for a guinea pig, for you to try this.’ I understand they want to tweak exercises… but there's tweaking and there's trying a completely different thing and going in a completely different direction, and that's what it felt like a lot.” [Female, 40-59 yr, 1 yr post stroke].

The extent to which participants would engage in rehabilitation on any given day differed. Participants' experiences ranged across a spectrum, from not attending rehabilitation at all, to being present but skipping activities, to going through the motions of the exercises without injecting effort or heart. However, it was almost universally acknowledged by participants that ‘no one else is going to do it for you.’

“You followed everything through because that's what you had to do. You didn't try to get out of it the easy way, whether you got up in the morning and didn't feel like going, you still done it because that's what you had to do.” [Female, 60 yr and over, 9 yrs. post stroke].

When it came to taking RPDs, some participants said that if it helped them, they would take it no matter the downside. Conversely, some participants said they would only tolerate so much.

“And that stage in the game I was in so much pain I would've taken bloody rabbit pellets if it worked.” [Female, 60 yr and over, 1 yr post stroke].

“It's a bit of a stretch to get up four o'clock in the morning every day [to take a tablet] and within two months I'm just the same.” [Female, 60 yr and over, 22 yrs. post stroke].

*Both decision points: Factors which influenced decisions at both decision points.*

*‘The people my rehabilitation and RPDs affect.’*

Participants often made decisions for and because of others in their life at both decision points. In some cases, participants described making decisions because it was beneficial to another person. Some were motivated because they wanted to recover for those they cared about.

Family members were often involved in decision making, especially when transport and finances were considerations. When resources like money or vehicles were shared, and the spouse was the main income earner or responsible for transporting the participant, this was important. Additionally, some participants described the impact of their stroke on their partner, feeling that their partner's life had been irrevocably changed.

“I think you've got to realize that you've got a loving partner….who's with you and going through this. It's just as hard on them…They suffer along with you.” [Female, 60 yr and over, 22 yrs. post stroke].

Guilt felt by participants who believed their stroke disrupted their family's lives was compounded by family members' dedication and commitment to participants' recovery.

“I'd like to get better anyway but my (*sic)* mainly for her. I'd rather do it for her than just for me. She's your loved one… she has to put up [with] a lot.” [Male, 60 yr and over, 3 yrs. post stroke].

Participants who enjoyed strong relationships with their adult children valued their input, considering their opinions when making decisions about rehabilitation. Some participants relied on their adult children for transport, which impacted what rehabilitation they did and when they could do it. Others were concerned about relying on their adult children too much.

“… he came home and he looked after me for two years. And I thought, ‘No. You're starting to resent me. I can feel it.’ … I said, ‘Look, I'm going to get help. I want you to go out and get a job and get a life.’” [Female, 60 yr and over, 4 yrs. post stroke].

The relationship between the participant and clinician played a role in participants' decision making. These clinicians were rehabilitation therapists and assistants, doctors, nurses, and pharmacists. Respect for and trust in their clinicians prompted participants to agree to the rehabilitation prescribed for them. Participants felt this trust was undermined when clinicians or their rehabilitation plan continued changing without consultation. These situations influenced how much they applied themselves during the rehabilitation session or sometimes whether they would return. However, some participants described being motivated by the obligation to attend appointments and didn't want to cancel; or because they had prepaid, or because it was polite and respectful to follow through.

Other participants said they took their prescribed medications because their ‘doctor said so’.

“I think there's a bit of blind trust, with, with doctor…Doctor says, ‘Oh, this does X Y Z and I will prescribe for you this *(sic)*.’” [Female, 40-59 yr, 7 yrs. post stroke].

*‘Fitting rehabilitation and RPDs into my life.’*

How rehabilitation would fit into the participants' and their family's daily schedule influenced participants' decisions about what rehabilitation to do. Timing and changing of times, energy levels and need for rest, and being able to negotiate the environment around them all came into play.

“I get invited somewhere, well, then, I've got to research it. What's the gutter look like, what's the footpath look like, how can I get in, can I go to the toilet? …Those [rehabilitation] timetable changes would just kill you…Because you're already trying to organise your support worker in the morning… and then transport, and then there's the knock-on effect of rearranging everything too.” [Female, 40-59 yr, 1 yr post stroke].

Participants discussed how the phase of their life impacted their decision-making. Whether they saw themselves as a student, a worker, a family-maker, or retiree – participants would make decisions about potential rehabilitation and RPDs with this in mind, and were prepared to go to great lengths.

“…There's no limit to what I would pay. … I would use up all my super[annuation]… One of my ultimate dreams is to go back to work…” [Male, 40-59 yr, 3 yrs. post stroke].

On having a stroke in their early twenties:

“[If] I had to be away … from my children for a significant period of time – it would affect the decision-making because I've got dependents… I would have to think about what I would do with my family if I had to leave them for an extended period of time.” [Male, under 40 yr, 14 yrs. post stroke].

Participating in the right amount of rehabilitation for their stage of recovery, especially the early stages of recovery, was important to participants. Where rehabilitation of higher intensity or frequency was available, participants described wanting to take advantage of windows of opportunity, with the understanding that it would increase neuroplasticity, which would lead to a better recovery.

…You have this window of opportunity now. If you don't do that, you don't get that chance again.” [Female, 40-59 yr, 7 yrs. post stroke].

Participants talked about the negatives and positives of taking RPDs, however these factors were of varied importance within and between participants. Participants discussed trade-offs between possible recovery outcomes and perceived drawbacks.

“I probably wouldn't like it, but if I knew it was going to prevent something or help something, I'd take it.” [Female, 40-59 yr, 1 yr post stroke].

“I don't think so…. Because I'm already taking enough medicines.” [Female, 40-59 yr, 8 yrs. post stroke].

## 4 Discussion

The factors that were identified to influence stroke survivors' decision-making about their rehabilitation and the prospect of taking RPDs to improve recovery, and the six themes within which they could be categorised, were not dissimilar to the factors discussed in the broader literature.^1^^,^^2^^,^^24^, ^25^, ^26^, ^27^ The unique finding in this study was that there were two distinct decision points for stroke survivors during the recovery process and there were factors to be considered at either one or both of those points:1) overall, when deciding the type of rehabilitation or RPD for stroke recovery; and 2) on a daily basis, when deciding to participate in rehabilitation and take RPDs on any given day. The importance placed on each factor varied within and between participants; consequently, the ultimate reason for each rehabilitation and RPD choice was unique.

Recognizing the two important decision points along the recovery trajectory provides a new perspective. The focus of previous qualitative stroke recovery studies has been on factors that constitute ideal therapy for stroke survivors, and the facilitators and barriers to their participation in their rehabilitation.^1^^,^^2^^,^^24^, ^25^, ^26^, ^27^ Discussing these factors at every decision point may work to leverage the stroke survivor's motivation to a) commit to rehabilitation initially, and b) actively engage at every rehabilitation session. Therapists need to be mindful of factors within their control (e.g., stroke survivors' recovery goals) so they may be able to influence the stroke survivors' decision to engage or not. Furthermore, decisions made at one decision point are likely to influence those made at the other decision point. Reminding stroke survivors of the trade-offs they have made and that have motivated their full participation in previous therapy sessions could encourage them to give their best efforts when drive and energy are lacking. Consequently, decisions made at one point should be considered with the other decision point in mind, to coach the stroke survivor to achieve optimal outcomes for their circumstance.

Factors influencing stroke survivors' decision-making about rehabilitation and RPDs will be relevant when investigating RPDs in the future. The best available evidence suggests that RPDs must be paired with physical or behavioural interventions to drive activity-dependent biological processes associated with stroke recovery.^28^^,^^29^ Thus, stroke survivors will need to participate in the *entire* stroke rehabilitation process if they are to gain any potential benefits of RPDs. The recovery pathway stroke survivors choose and how they engage with rehabilitation daily requires consideration of factors for both physical and behavioural interventions and RPDs. Committing to RPDs requires commitment to the physical or behavioural intervention, as RPDs do not currently appear to lead to stroke recovery on their own. Therefore, considering factors for RPDs along with physical or behavioural interventions when deciding which stroke recovery process to undertake is more likely to lead to success.

While the outcome of this study offers new and valuable insights into factors affecting stroke survivors' decision-making regarding their rehabilitation or taking RPDs, several methodological limitations must be discussed to place these findings within perspective. Firstly, as all study participants volunteered to attend, findings may reflect views of those for whom recovery is a priority.^30^ Alternatively, these participants may have been motivated by either mostly positive or mostly negative experiences, and findings may therefore represent extremes of the stroke survivor experience. Stroke survivors who cannot or did not choose to participate in this study may have different views. Secondly, most participants were living in regional communities at the time of their stroke and recovery. This study will reflect views of Australian stroke survivors living outside of metropolitan areas where rehabilitation options are limited compared to larger cities.^31^

Insights gained from this study can aid development of discrete choice experiments investigating stroke survivors' preferences for RPDs as part of a rehabilitation program. To date, such investigations have not been undertaken as RPDs are still being trialed. To illustrate, a discrete choice experiment could be conducted during protocol development for a trial investigating the benefits of taking a candidate RPD in conjunction with task-specific training. Determining the most favourable combination of attributes for the RPD dosing regime and task-specific training sessions, based on participants' preferences, may lend itself to higher rates of participation and adherence to the trial protocol, which in turn may lead to more robust results.

Investigating factors which influence stroke survivors' decision-making about rehabilitation and RPDs has highlighted two important decision points. Acknowledging these critical decision points means stroke survivors can be supported in those moments to make the best decisions for themselves to participate in rehabilitation and take RPDs to achieve their full recovery potential. RPDs alone have not been shown to be effective; future investigations into their efficacy must include a physical or behavioural intervention component. To achieve optimal recovery with a combined RPD and rehabilitation intervention, stroke survivors will need to adhere to both components. Poor adherence to any stroke rehabilitation intervention will deliver underwhelming outcomes. The findings highlight the importance of not making assumptions about why stroke survivors' make decisions about their rehabilitation or RPDs, but rather understanding the interplay between factors which influence their decisions. Understanding these factors and exploiting them in the design of person-centred rehabilitation and RPD interventions, will see higher engagement and increased recovery outcomes.

## Author contributions

NF conceived the research question and design of the interview topic guide. NF and RR analysed the data. NF and RB interpreted the data. All authors were involved in the writing and critical revision of the manuscript. All authors have read and approved the manuscript.

## Declaration of Competing Interest

No potential conflict of interest was reported by the authors.

## Appendix A Supplementary data

Supplementary material Image 1
