# Supplementary material for: Stroke survivors' perspectives on decision-making about rehabilitation and the prospect of taking recovery-promoting drugs: A qualitative study
Source: Explor Res Clin Soc Pharm. 2023 Jun 28;11:100297. doi: 10.1016/j.rcsop.2023.100297 (PMC10338145; doi:10.1016/j.rcsop.2023.100297)
Supplement: Supplementary file 1 — Supplementary material [file mmc1.docx]

| **Table S1. Interview topic guide** | |
| --- | --- |
| **Domain** | **Topic Questions and Probes** |
| Introduction | *Question*: Let’s start by going around the group - could you please introduce yourselves, and briefly share your experience of rehabilitation? |
| Rehabilitation after stroke:   - Inpatient | *Question*: For those of you who undertook rehabilitation as inpatients, what made it worthwhile? Why?  *Probe*: ‘The things that made it easier to tolerate the drawbacks’  *Follow-up question:* What made it harder? Why?  *Probe*: ‘What if you found the exercise boring, would you keep going?’  *Follow-up question:* What would make you stop participating in inpatient rehabilitation, or put in less than your best effort? Why?  *Probe*: ‘What if you didn’t like the therapist, would you keep going?’ |
| Rehabilitation after stroke:   - Outpatient | *Question*: When doing rehabilitation outside of hospital, what made it worthwhile for you? Why?  *Probe*: ‘The things that made it easier to tolerate the drawbacks’  *Follow-up question:* What made it harder to do rehabilitation, or harder to go to rehabilitation? Why?  *Probe*: ‘Would travel time to the rehabilitation centre/clinic make a difference to you?’  *Follow-up question:* What would make you stop going to rehabilitation, or put in less than your best effort? Why?  *Probe*: ‘What kind of cost for rehabilitation would make a difference to you, whether you went or how hard you tried in your rehabilitation sessions?’ |
| Taking pharmaceutical treatments | *Question*: What’s important to you when deciding whether to start a new medication?  *Probe*: The important things that you might ask a doctor or a pharmacist about, before deciding whether it is worth taking the medicine.  *Follow-up question:* Are there drawbacks of a medication that you would put up with if it meant you would have a better response with your stroke rehabilitation (arm rehabilitation)?  *Follow-up question:* How much benefit (i.e. what would it look like for you) would a medication have to have on your arm rehabilitation for you to put up with the medication drawbacks? |
| Getting advice when making these decisions | *Question*: Who else’s opinions matter to you when are making decisions about rehabilitation? |
| Closing | *Question:* We are now approaching the end of our discussion. Is there anything else anyone would like to add? |
